# Effectiveness of strategies to encourage general practitioners to accept an offer of free access to online evidence-based information: a randomised controlled trial

**DOI:** 10.1186/1748-5908-4-68

**Published:** 2009-10-20

**Authors:** Heather Buchan, Emma Lourey, Catherine D'Este, Rob Sanson-Fisher

**Affiliations:** 1 National Health and Medical Research Council, Melbourne, Australia; 2 The University of Newcastle, Newcastle, Australia; 3 School of Medicine and Public Health, The University of Newcastle, Newcastle, Australia

## Abstract

**Background:**

This study examined the effectiveness of seven different interventions designed to increase the proportion of general practitioners (GPs) accepting an offer of free access to an online evidence-based resource.

**Methods:**

Australian GPs (n = 14,000) were randomly selected and assigned to seven intervention groups, with each receiving a different letter. Seven different strategies were used to encourage GPs to accept an offer of two years free access to an online evidence-based resource (BMJ *Clinical Evidence*). The first group received a standard letter of offer with no experimental demands. Groups two to seven received a standard letter of offer outlining the requirements of the study. They were asked to complete an initial online questionnaire, agree to complete a 12-month follow-up questionnaire, and agree to having data about their usage of the online evidence-based resource provided to researchers. Groups three to seven also had additional interventions included in the letter of offer: access to an online tutorial in use of the resource (group three); provision of a pamphlet with statements from influential opinion leaders endorsing the resource (group four); offer of eligibility to receive professional development points (group five); offer of eligibility for a prize of $500 for registration at a medical conference of their choice (group six); and a combination of some of the above interventions (group seven).

**Results:**

In the group with no research demands, 27% accepted the offer. Average acceptance across all other groups was 10%. There was no advantage in using additional strategies such as financial incentives, opinion leader support, offer of professional development points, or an educational aid over a standard letter of offer to increase acceptance rates.

**Conclusion:**

This study showed low acceptance rates of the offer of access to the online resource when there was an associated requirement of response to a short online questionnaire and non-obtrusive monitoring of GP behaviour in terms of accessing the resource. If we are to improve care and encourage evidence-based practice, we need to find effective ways of motivating doctors and other health professionals to take part in research that can inform our implementation efforts.

## Background

Access to high quality evidence-based resources is a necessary first step if doctors are to change clinical practices in line with best available evidence [1-3]. The rapid speed of scientific research brings daily breakthroughs. An extensive review of health and medical journal articles published in Medline between 1978 and 2001 revealed that by 2001 the average number of articles published per year was 442,756 [4]. Clinicians not only lack the time to locate and review such extensive numbers of journal articles, but many also lack skills necessary for locating them [5,6]. Even when relevant literature is located, clinical research does not always easily translate into practical advice for clinicians [7]. Given the volume of research produced and the skills required to locate and interpret relevant current evidence, it is apparent that research knowledge needs to be synthesised into a practical and accessible format for clinicians. Over recent years, electronic methods have increasingly been used to provide this kind of information to clinicians, and a number of countries have invested in national licenses for various clinical resources.

One resource that claims to assist clinicians in overcoming the barriers to finding and reviewing best evidence is BMJ *Clinical Evidence*. This is available online and provides summaries about the prevention and treatment of selected clinical conditions commonly seen in primary and hospital care settings. These summaries of conditions are produced using comprehensive reviews and evaluations of the literature [8].

In Australia, the National Institute of Clinical Studies (NICS), now an institute of the National Health and Medical Research Council (NHMRC), was established to improve health care by getting the best available evidence from health and medical research into everyday practice. As part of its brief to make evidence more accessible to clinicians, the institute undertook a study, funded by the Australian government, to examine the acceptance by Australian general practitioners (GPs) of an offer of free access to the online version of BMJ *Clinical Evidence *and its subsequent use. A number of general practice leaders and organisations had strongly advocated that this resource should be freely available to GPs. The cost of a single user 12-month subscription is approximately $300AUD. Participants in the study were offered free access to the resource for two years.

Not all doctors offered access to an evidence-based resource will be interested in accepting or using the resource. We wanted to investigate whether any particular strategy would encourage doctors to accept this offer of free access. The objectives of this study were to:

1. Examine the effectiveness of different strategies designed to encourage GPs to accept an offer of free access to an online evidence-based resource.

2. Compare the characteristics of those who accepted the offer and those who did not.

## Methods

### Participants

Participants were randomly selected by Medicare Australia, the Australian government agency responsible for processing claims and reimbursements to the public for visits made to GPs. At the time the study was undertaken, there were 22,996 doctors listed by Medicare Australia as providing general practice services. Of these, 18,262 doctors were deemed eligible for participation in the study on the basis that they were classified by Medicare as being in active practice (having the primary speciality of general practice and making at least $1000 of Medicare claims in the preceding quarter). The socio-demographic characteristics of these GPs are shown in Table 1. From this group, a random sample of 14,000 GPs was selected and randomly allocated using computer-generated randomisation to one of seven groups, stratified by age group, gender, and location, as determined by the Accessibility/Remoteness Index of Australia (ARIA) [9].

**Table 1:** Socio-demographic characteristics total eligible population

	**18,262**	
	
**Age**	**N**	**%**
<35	1,578	8.6

35 to 44	4,548	25

45 to 54	6,160	34

55 to 64	4,355	24

65 +	1,621	8.9

**Gender**		

Female	6,779	37

Male	11,462	63

**Country of Graduation**		

Australia	13,369	73

UK/Ireland	1,382	7.6

Asia	1,750	9.6

Europe	406	2.2

Africa	761	4.2

Other	594	3.3

**Years since graduation**		

<5	268	1.5

5 to 9	1,478	8.1

10 to 19	4,629	25

20 to 29	6,450	35

30 to 39	3,953	22

40 +	1,484	8.1

**ARIA Classification**		

Highly Accessible	15,292	84

Accessible	1,805	9.9

Mod Accessible	538	2.9

Remote	144	0.8

Very Remote	135	0.7

Some data missing for gender and ARIA classification. Percentages may not add to 100 due to rounding.

### Procedure

Medicare Australia forwarded an invitation letter from NICS to the selected GPs offering two years free access to the online version of BMJ *Clinical Evidence*. Using Medicare for this process ensured complete coverage of the GP population as it possesses the most accurate, current, and reliable contact information on Australian GPs due to its role in processing claims and payments to GPs.

The letter stated Medicare Australia would provide NICS with de-identified grouped data on the characteristics of those GPs who accepted the offer and of those who rejected or didn't respond to the offer. Groups two to seven received letters which indicated that if GPs accepted they would be asked to complete an initial online survey and a subsequent 12-month follow-up survey; and to consent to NICS receiving information about their use of the online evidence-based resource from the publishers. They were assured that individual practitioners would not be identified in any reports or publications arising from the study. The requirement for completion of the online questionnaires and agreement to usage monitoring were for a companion study of perceptions and usage of the online resource.

All seven groups were given four weeks to return the consent form, via a reply paid envelope or fax. GPs in group one who returned their consent form within four weeks were eligible for inclusion in the study. GPs in groups two to seven who returned their agreement form within four weeks and completed the online survey by the specified date were eligible to participate in the study. Non-responders did not receive reminders during this four-week period.

Once the acceptance form was returned, GPs received a confirmation email specifying a date they would receive their account details to log onto the online evidence-based resource. Confirmation emails sent to groups two to seven contained additional instructions on how to complete the online survey. Personalised reminder emails were sent to GPs who had not completed the questionnaire. All GPs eligible to participate were emailed their account details to the online evidence-based resource with instructions on how to access the site.

### Interventions

Each intervention was specifically designed, based on current literature, to encourage GPs to accept the offer and participate in the study. The interventions were also designed to be practical and cost effective options that could be replicated by other researchers interested in undertaking studies with health practitioners.

### Group one: No experimental demands

This group was offered two years of free online access to the evidence-based resource, and was only required to return the consent form to be eligible. They were not required to consent to their individual usage data of the resource being released for analysis. This groupallows the uptake rate, without any associated experimental requests, to be examined.

### Group two: Standard invitation

This group was offered two years of online free access, provided they completed an online questionnaire, agreed to complete a 12-month survey, and allow data about their usage of the resource to be provided to the researchers. Comparisons between group one and two provided an opportunity to evaluate the effect that study demands had on response rate.

### Group three: Tutorial

Although the integration of computers into general practice has increased considerably over the last decade, barriers to their use still exist, with many GPs still lacking confidence, skills, training, and technical support [10-12]. In an effort to reduce technical barriers, group three was offered access to a short, downloadable tutorial specifically developed by NICS to demonstrate how to access and search the site of the evidence-based resource. The NICS' online tutorial consists of an interactive flash movie and requires Adobe flash player 8. Tutorial topics include instructions on how to login, search for keywords, search for frequently searched conditions, print, use help option, contact the publishers, access resources, update details, and log out.

### Group four: Opinion leaders

Literature is mixed as to whether using opinion leaders to endorse evidence-based decision aids can improve uptake [13-15]. Group four received a pamphlet containing supportive statements regarding the benefits of the online evidence-based resource from leaders of various Australian general practice and medical organisations. A variety of well known opinion leaders were used in an attempt to overcome difficulties in clearly identifying individuals and organisations that might be perceived as influential by a majority of the selected GPs. Statements made included:

'As a rural or remote medical practitioner you often have to manage complex conditions without nearby specialist support. *Clinical Evidence *provides some of the very best evidence-based support for you in an electronic format.'

'*Clinical Evidence *is a trusted source of summarised evidence-based clinical information that is presented in an easy to read format. It provides clinicians with answers to many of the important questions which arise during our consultations.'

'As a GP and educator, I face questions every day. Patients and learners have questions. I have questions. I see *Clinical Evidence *as a great resource to improve the quality of the answers we find.'

### Group five: Acquisition of professional development points

To maintain access to certain Medicare payments, every triennium GPs must earn 130 Royal Australian College of General Practitioners (RACGP) professional development points (undertaking a minimum of two Category one activities) or 100 Australian College of Rural and Remote Medicine (ACRRM) professional development points. GPs can gain these points through a range of activities, with the category one activities generally being more time intensive and therefore worth more points. Consequently, offering professional development points to GPs for their participation in an activity might increase GP involvement. Group five was offered eligibility to earn 30 Category one points through the RACGP or 20 points through the ACRRM. To receive these points, GPs were required to develop learning objectives, regularly use the online resource for a 12-month period, and then complete a survey about the extent to which they met their learning objectives. Doctors in this group offered the opportunity to gain CPD points did not have to take up this offer in order to get the resource.

### Group six: Eligibility for a prize

Various types of monetary incentives are widely used by pharmaceutical companies to recruit GPs to studies; such incentives may also increase the uptake of education material and improve response rates in mailed questionnaires [16-19]. Members of the sixth group were informed that doctors who agreed to participate would be eligible for a prize of $500 towards registration for a medical conference of their choice.

### Group seven: Combination intervention

Some studies have shown that multifaceted interventions are more effective than single interventions when encouraging clinicians to use evidence [20,21]. Group seven received a combination of interventions comprising of the opinion leaders' pamphlet, access to the online tutorial and eligibility to earn professional development points through participating in the study.

Access to the online evidence-based resource was not dependent upon GP's use of incentives offered. For example, GPs offered access to an online tutorial did not have to use it in order to gain free access to the online evidence-based resource.

### Statistical methods

Data on response status by intervention group and by age, gender, country of graduation, years since graduation, and Accessibility/Remoteness Index of Australia (ARIA) were provided by Medicare in table format (to protect GP's privacy).

Baseline characteristics (age group, gender, country of graduation, years since graduation, and ARIA) of all doctors selected for inclusion in the study were compared between intervention groups. To investigate factors associated with acceptance of the offer, response rates were compared between intervention groups, and between levels of socio-demographic variables. Because of small numbers in some cells and/or the large number of categories, where appropriate, some categories of age, years since graduation, and ARIA were combined. To determine whether any differences in characteristics associated with uptake translated into differences in characteristics of responders, we compared factors between groups for those GPs who responded. All comparisons were undertaken using the chi-square test (as all variables were categorical).

It was anticipated that 10% to 50% of GPs would take up the offer of free access to an online evidence-based resource. A sample of 2,000 per group (14,000 in total) would allow a detectable difference in response rates between groups, and in characteristics between responders versus non-responders of 3% to 7%, depending on response rates (assuming 80% power and 5% significance level).

### Ethical approval

Ethics approval was given by the Royal Australian Collage of General Practitioner's National Research and Evaluation Ethics Committee.

## Results

**Figure 1:**
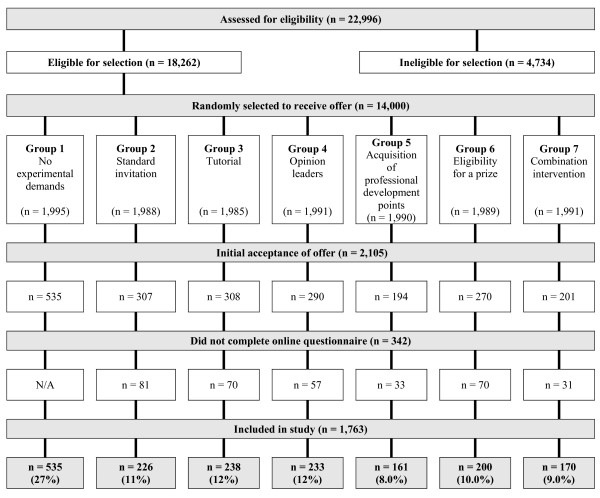
**Flow of participants through the study**. Note: initial numbers in groups may not total to 2,000 each as there were 71 return to senders recorded by Medicare.

Age, gender, country of graduation, years since graduation and area of residence were similar among the seven intervention groups. Of the 14,000 letters mailed, Medicare reported that 71 letters were return to sender (0.5%). There were 2,105 (15%) signed acceptance forms returned. Of the 1,570 GPs assigned to groups two to seven who accepted, 1,228 went on to complete the online questionnaire, which when combined with the 535 GPs assigned to group one who accepted, gives a final acceptance rate of 12.5% (n = 1763). There was a statistically significant difference in acceptance among the groups, with acceptance highest in group one (no experimental demands) (27%), and lowest in group five (offer of professional development points) (8.0%) and group seven (combined interventions) (8.5%). Acceptance rates were similar for groups two to seven ranging from 8.0% to 12% (Figure 1).

Given the large sample size, there was adequate power to detect small differences in socio-demographic characteristics between responders and non-responders. Relative to non-responders, responders were more likely to be younger, male, to have graduated in Australia, UK, or Ireland, to have graduated more recently and practice in a highly accessible geographic location. For those GPs who responded, there were no statistically significant differences in age, gender, country of graduation, or ARIA classification among the groups, while time since graduation varied among the intervention groups (data not shown).

## Discussion

In this study, acceptance of a free online information resource (that would usually require payment for access and that had been identified by a number of GP groups as potentially useful and valuable) was significantly lower among groups asked to complete online questionnaires and consent to usage data being monitored compared to the group with no experimental demands.

All groups offered the resource needed to make some effort to respond -- they were required to complete and return an acceptance form by mail or fax so that they could be registered to log on to the resource. Twenty-seven percent of the doctors in group one, who received a letter offering the resource without the need for participation in the companion study, accepted. In contrast, on average only 10% of doctors offered free access in return for participation in the companion study accepted this offer. The additional demands placed on doctors in groups two to seven relating to the companion research into perceptions of usage of the resource, completion of the online questionnaire, and monitoring of usage appears to have been a significant disincentive to acceptance of the resource.

Low cost strategies designed to provide additional incentives to participate in the companion study (such as endorsement by opinion leaders) or reduce barriers to acceptance (such as offer of an online tutorial in use of the resource) were no more effective than a standard letter of offer.

The differences in characteristics of doctors responding to the offer and those not responding may reflect more the attractiveness of the offer of an online resource than willingness to participate in the research. We hypothesised that doctors in rural areas of Australia would be less likely to accept the offer due to limited broadband access, however research indicates rural GPs are more likely to access the internet despite having poorer access [22]. Younger doctors are more likely to be interested in an online resource than those who are older [23,24].

This is a very large population study investigating the effectiveness of different strategies designed to encourage GPs to accept an offer of free access to an online evidence-based resource and to participate in a study of its use and value. The study provided 14,000 GPs with the opportunity to access an online evidence-based resource at no financial cost to them. The strategies used in an attempt to encourage participation were low cost and could be used by researchers or other organisations interested in recruiting GPs to studies or encouraging GP uptake of a variety of resources. The collaboration with Medicare Australia provided information that would otherwise be unobtainable on non-responders to the letter of offer.

There were some limitations to this study. Doctors were only approached by letter which, because of privacy concerns, was not sent directly from researchers but forwarded by Medicare Australia, the government agency responsible for processing claims for GP reimbursement. This method of approach may have influenced acceptance of the offer, with some doctors possibly perceiving there to be a risk of data being shared with the same organisations responsible for processing GP reimbursements.

Non-participation may be attributable to factors other than aversion to online evidence-based resources, such as not liking the particular resource on offer or due to the additional burden of participation. Doctors may also have failed to respond to the offer for a number of other reasons, including lack of willingness to respond to unsolicited mail, because of a general dislike of unsolicited mail, or because the resource was unattractive to them. These factors may also have had different impact in different socio-demographic groups, given the differences we noted between responders and non responders.

There would have been some contamination of the sample, with some doctors within the same practice receiving different letters of offer. The offer of acquisition of professional development points for participation in the study (groups five and seven) was made halfway through the 2005 to 2007 triennium when many GPs may have already acquired the compulsory number of points. Because doctors in group one were not required either to complete online surveys or to agree to their usage of the resource being monitored, we do not know whether there would be a difference in doctors' willingness to participate if only one of these requirements was in place.

The study provides baseline data on what level of acceptance can be expected when offering GPs a free resource in return for participation in a companion study that requires them to respond to an online questionnaire and to agree to information about non obtrusive monitoring of their behaviour. Additional low-cost incentives, including offer of continuing education points, opinion leader endorsement, offer of an online tutorial or offer of entry into a lottery for money to be used on conference attendance made no difference to acceptance of the offer.

## Conclusion

While this study was based on an Australian GP population, the findings have general implications for researchers, medical educators, and policymakers. Funding of universal access to free online resources may not be cost-effective if calculations of cost are based on total population eligible to use the resource rather than the much smaller number likely to be interested.

It is of critical importance to find ways of increasing the probability that GPs will access information regarding best evidence practice. Unless GPs access best evidence resources, there is little chance that they will read them and potentially change their clinical practice. Getting them to agree to access is a first necessary step.

To understand how to improve practice, we need to be able to engage health professionals in research about changing behaviour [25]. Despite the offer of a free resource worth about $600 (for two years access) only 10% of doctors were willing to accept the resource when required to participate in a companion study of their use of the resource and its perceived value to them. GPs are often asked to recruit their patients to studies but are less frequently asked to participate in studies of their own behaviour. Patients who participated in research are motivated by a variety of factors, from altruism -- the belief that others may benefit from the knowledge gained--to hope that participation in research will improve the care they receive and favourably influence their outcome [26]. If we are to improve care and encourage evidence-based practice, we need to find equally effective ways of motivating doctors and other health professionals to take part in research that can inform our implementation efforts.

## Competing interests

The authors declare that they have no competing interests.

## Authors' contributions

HB obtained funding for the study, prepared ethics applications, contributed to the design of study, data analysis and interpretation, and writing of paper. CD undertook statistical analysis and contributed to writing the results section. EL contributed to writing of paper, project management, data management, ethics amendments, progress and final reports, development of online questionnaires, contributed to data analysis, and interpretation. RSF was responsible for design of study, and contributed to data analysis, interpretation, and writing of paper. All authors acknowledge that they have approved the final version of the paper submitted.

## References

[B1] Barton S (2001). Using clinical evidence. British Medical Journal.

[B2] Straus S (2005). Teaching evidence-based medicine skills can change practice in a community hospital. The Journal of General Internal Medicine.

[B3] Glasziou P (2006). Managing the evidence flood. Surgical Clinics of North America.

[B4] Druss B, Marcus S (2005). Growth and decentralization of the medical literature: Implications for evidence-based medicine. Journal of the Medical Library Association.

[B5] McColl A (1998). General practitioners' perceptions of the route to evidence based medicine: A questionnaire. British Medical Journal.

[B6] Oliveri R, Gluud C, Wille-Jørgensen P (2004). Hospital doctors' self-rated skills in and use of evidence-based medicine: A questionnaire survey. Journal of Evaluation in Clinical Practice.

[B7] Tonelli M (2001). The limits of evidence-based medicine. Respiratory Care.

[B8] (2007). BMJ Clinical Evidence [homepage on the Internet].

[B9] Department of Health and Aged Care. Accessibility/Remoteness Index of Australia (ARIA). Canberra: The Department, October 2001. (Occasional Papers Series No. 14). http://www.health.gov.au/internet/main/publishing.nsf/Content/health-historicpubs-hfsocc-ocpanew14a.htm.

[B10] Henderson J, Britt H, Miller G (2006). Extent and utilisation of computerisation in Australian general practice. Medical Journal of Australia.

[B11] Keddie Z, Jones R (2005). Information communication technology in general practice: Cross sectional survey in London. Informatics in Primary Care.

[B12] Janes R (2005). Rural New Zealand health professionals' perceived barriers to greater use of the internet for learning. Rural and Remote Health.

[B13] Heywood A (1995). Reducing systematic bias in studies of general practitioners: The use of a medical peer in the recruitment of general practitioners in research. Family Practice.

[B14] Bhandari M (2003). A randomized trial of opinion leader endorsement in a survey of orthopaedic surgeons: Effect on primary response rates. International Journal of Epidemiological Association.

[B15] Thomson O'Brien M (1999). Local opinion leaders: Effects on professional practice and health care outcomes (review). The Cochrane Database of Systematic Reviews.

[B16] Blumenthal D (2004). Doctors and drug companies. The New England Journal of Medicine.

[B17] Lemmens T, Miller P (2006). Regulating the market in human research participants. PLoS Med.

[B18] Foy R (2003). How evidence based are recruitment strategies to randomized controlled trials in primary care?. Experience from seven studies.

[B19] Edwards P (2005). Meta-analysis of randomised trials of monetary incentives and response to mailed questionnaires. Journal of Epidemiology and Community Health.

[B20] Wensing M, Weijden T van der, Grol R (1998). Implementing guidelines and innovations in general practice: Which interventions are effective?. British Journal of General Practice.

[B21] Chaillet N (2006). Evidence-based strategies for implementing guidelines in obstetrics: A systematic review. Obstetrics & Gynaecology.

[B22] Britt H (2003). General practice activity in Australia 2002-03. AIHW Cat. No. GEP 14.

[B23] Martin S (2004). Younger physicians, specialists use Internet more. Canadian Medical Association Journal.

[B24] Gjersvik PJ, Nylenna M, Aasland O (2002). Use of the Internet among dermatologists in the United Kingdom, Sweden and Norway. Dermatology Online Journal.

[B25] Grol R, Grimshaw J (1999). Evidence based implementation of evidence based medicine. The Joint Commission Journal on Quality and Improvement.

[B26] Wright J (2004). Why Cancer Patients Enter Randomized Clinical Trials: Exploring the Factors That Influence Their Decision. Journal of Clinical Oncology.

